# HIV-1 Fusion Inhibitor Peptides Enfuvirtide and T-1249 Interact with Erythrocyte and Lymphocyte Membranes

**DOI:** 10.1371/journal.pone.0009830

**Published:** 2010-03-23

**Authors:** Pedro M. Matos, Miguel A. R. B. Castanho, Nuno C. Santos

**Affiliations:** Instituto de Medicina Molecular, Faculdade de Medicina da Universidade de Lisboa, Lisboa, Portugal; Tsinghua University, China

## Abstract

Enfuvirtide and T-1249 are two HIV-1 fusion inhibitor peptides that bind to gp41 and prevent its fusogenic conformation, inhibiting viral entry into host cells. Previous studies established the relative preferences of these peptides for membrane model systems of defined lipid compositions. We aimed to understand the interaction of these peptides with the membranes of erythrocytes and peripheral blood mononuclear cells. The peptide behavior toward cell membranes was followed by di-8-ANEPPS fluorescence, a lipophilic probe sensitive to the changes in membrane dipole potential. We observed a fusion inhibitor concentration-dependent decrease on the membrane dipole potential. Quantitative analysis showed that T-1249 has an approximately eight-fold higher affinity towards cells, when compared with enfuvirtide. We also compared the binding towards di-8-ANEPPS labeled lipid vesicles that model cell membranes and obtained concordant results. We demonstrated the distinct enfuvirtide and T-1249 membranotropism for circulating blood cells, which can be translated to a feasible *in vivo* scenario. The enhanced interaction of T-1249 with cell membranes correlates with its higher efficacy, as it can increase and accelerate the drug binding to gp41 in its pre-fusion state.

## Introduction

The Human Immunodeficiency Virus type 1 (HIV-1) is a highly pathogenic, evasive and difficult to eradicate agent that causes Acquired Immunodeficiency Syndrome (AIDS). This discovery in the early 1980s triggered major international scientific efforts in antiviral drug discovery and development [1]. As a consequence, many drugs are now available to manage this condition, allowing the use of drug combination therapy known as HAART (highly active antiretroviral therapy). The majority of the drugs inhibit the different enzymes vital for the HIV-1 life cycle: reverse transcriptase, integrase and protease. However, the entry inhibitors target steps before the viral content is released into the host cell cytoplasm [2]. Infection of T CD4^+^ cells begins with the binding of the viral envelope trimeric glycoprotein gp120 with the CD4 receptor and a chemokine receptor (CCR5 or CXCR4) of the host cell. This engagement triggers the exposure of the hydrophobic N-terminal region of another envelope glycoprotein, gp41. This fusion peptide anchors to the membrane of the host cell, enabling the two gp41 helical heptad repeat domains, the C-terminal (CHR or HR2) and the N-terminal (NHR or HR1), to fold into each other to form a hairpin-like structure (6-helix bundle). This approximates the cell and the viral membranes, facilitating their fusion and the release of the viral content into the cell [3].

Besides maraviroc, a recently approved CCR5 antagonist blocking gp120 co-receptor engagement [4], enfuvirtide is the other only entry inhibitor approved for clinical use [5]. Enfuvirtide (formerly known as T-20 and DP-178) is a peptide drug selected from chemically synthesized peptides derived from various regions of gp41 [6]. The core structure of gp41 was only revealed later and helped to understand the inhibitory activity of enfuvirtide [7]. The peptide sequence (sequence 643–678 of HIV-1_LAI_ [6]) corresponded partially to the CHR region of gp41 and it would bind to the opposite NHR region, preventing the formation of the hairpin structure and consequently, the fusion. The same consortium that led enfuvirtide to clinical approval (Trimeris, Inc. and Roche) developed a second generation fusion inhibitor, T-1249. It is a 39-mer peptide which sequenced was designed taking into account the gp41 CHR sequences from HIV-1, HIV-2 and SIV (Simian Immunodeficiency Virus) [8]. A successful short-term evaluation of antiretroviral activity and safety in humans proved the potential of this new drug [8], although further clinical development was put on hold [9].

Since the first appearance of enfuvirtide, the search for peptide drugs against HIV has been a growing field of research and several candidates proved to be efficient *in vitro* [10]. As our knowledge of HIV structure and function progresses, more sophisticated peptide designs are developed in order to overcome viral resistance to previous drugs. The initial view of the mechanism of action of these peptides, that is, binding to NHR region and block 6-helix bundle formation, has been refined over the years. Other factors besides gp41-peptide binding can contribute to explain the mode of action of these peptides. In fact enfuvirtide does not form stable 6-helix bundle structures with N36, a NHR derived peptide, contrary to C34, another CHR derived peptide [11]. Moreover, peptides derived from the membrane-spanning domain in gp41 and the co-receptor binding site in gp120 inhibited enfuvirtide antiviral activity [11], meaning that other sites in the viral envelope could be targeted by enfuvirtide.

Membrane binding/tropism emerged as an important factor to take into account concerning the mode of actions of these peptides. Previous studies evaluated the preferences of enfuvirtide and T-1249 to membrane model systems. Partition of these peptides to fluid phase and cholesterol-rich lipid vesicles was followed by intrinsic tryptophan fluorescence present in both peptides [12], [13]. Both enfuvirtide [13] and T-1249 [12] partitioned to fluid phase zwitterionic phospholipid vesicles (POPC, 1-palmitoyl-2-oleoyl-*sn*-glycero-3-phosphocholine) with partition constants (*K*_p_ [14]) of 1.6×10^3^ and 5.1×10^3^, respectively. However, regarding interaction with cholesterol-rich POPC vesicles (up to 33% mol) enfuvirtide showed residual association, while T-1249 binding was significant, despite also presenting a low partition (membrane insertion) [12]. T-1249 improved preference for lipid environment correlated with its higher antiviral activity.

In this study, we aim to understand the nature of HIV-1 fusion inhibitors-membrane interactions using human blood cells, namely erythrocytes and PBMC (peripheral blood mononuclear cells). We made use of an indirect reporter, the fluorescent lipophilic probe di-8-ANEPPS, as direct measure of the peptide intrinsic tryptophan fluorescence was impracticable with cells. This probe incorporates in the outer leaflet of the cell membranes and is sensitive to the local dipole potential by shifting its excitation spectrum [15]. Interaction of peptides or other molecules with membranes can change the lipid and interfacial water dipoles organization. Moreover, the peptide dipoles themselves can contribute to the potential. These overall dipolar changes occurring at the membrane can then be translated by the probe both qualitatively and quantitatively [16].

## Results

### Labeling of erythrocytes and PBMC with di-8-ANEPPS membrane probe

**Figure 1:**
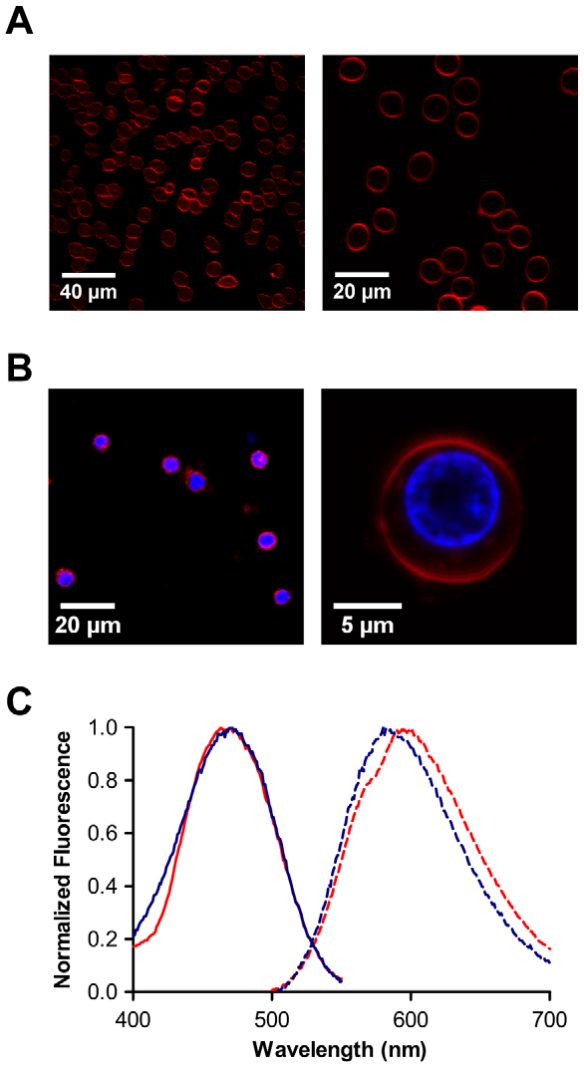
Visualization and spectral characterization of di-8-ANEPPS labeled cells. Labeled erythrocytes (A) and PBMC (B) were observed under a confocal fluorescence microscope to confirm that only the plasma membrane was stained (shown in red). Additional staining of the nucleus with Hoechst dye (blue) was performed for PBMC, only for microscopy observation. Excitation and emission spectra (C) were taken for each labeled cell type, differing on peaks due to different membrane environments (blue trace for PBMC and red trace for erythrocytes; filled line for excitation and dashed line for emission spectra). Excitation at 465 nm for both cell types; emission at 600 nm and 585 nm for erythrocytes and PBMC, respectively.

Isolated human erythrocytes and PBMC membranes were successfully labeled with di-8-ANEPPS. Confocal microscopy images clearly show the delineated labeled membrane (Fig. 1A,B). Importantly, no intracellular staining of di-8-ANEPPS was observed. The emission and excitation spectra of the probe were taken for the two cell types (Fig. 1C). Labeled erythrocytes and PBMC had different spectral maxima (specially for the emission) due to the probe sensitivity to the differences in the composition and nature of the membrane lipids.

### Enfuvirtide and T-1249 induce changes in the dipole potential of cell membranes

**Figure 2:**
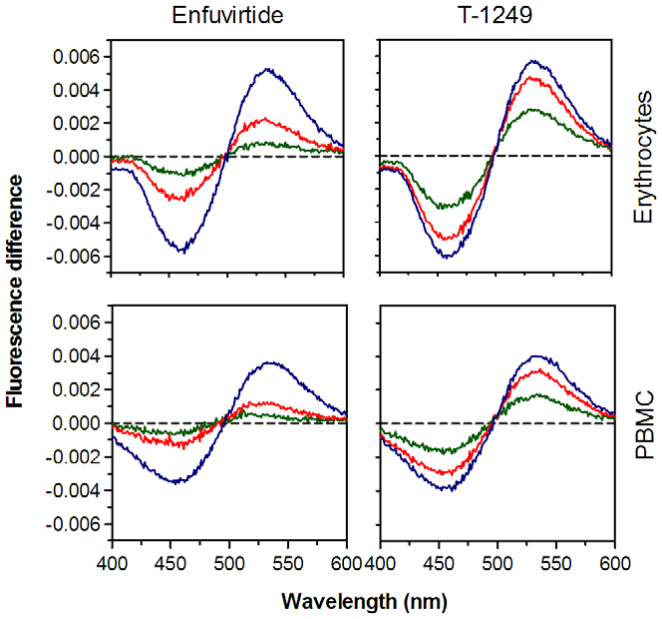
Differential spectra of membrane-bound di-8-ANEPPS in the presence of HIV-1 fusion inhibitor peptides. The shape of the difference spectra reveal spectral shift to the red, indicative of a decrease in membrane dipole potential. For erythrocytes (top row) and PBMC (bottom row), the higher the peptide concentration (150 µM in blue, 40 µM in red and 10 µM in green), the higher the spectral shifts.

Incubation of the labeled erythrocytes and PBMC with enfuvirtide or T-1249, lead to changes in spectral properties of the membrane-bound di-8-ANEPPS. Excitation spectra were deviated to the red meaning that a decrease in the dipole potential occurs. Differential spectra can be used in order to help the visualization of the spectral shift (Fig. 2). In this case, the minimum of the differential spectra comes at a lower wavelength than the maximum, indicative of a decrease in the dipole potential (see methods section). These changes depend on the peptide and its concentration. T-1249 is more effective in decreasing the membrane potential than enfuvirtide.

**Figure 3:**
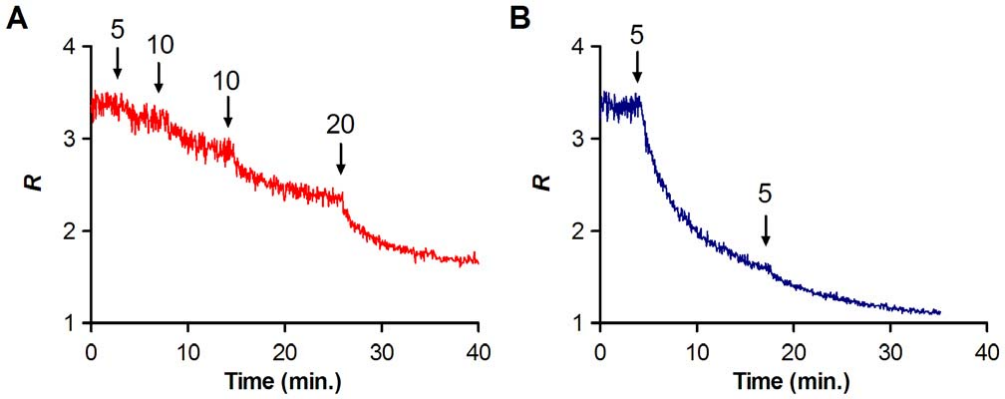
Time course variation of dipole potential in response to the peptides. The effect of the addition of indicated concentrations (µM) of enfuvirtide (A, red) and T-1249 (B, blue) to labeled erythrocytes was followed by measuring the ratio in real-time. Stepwise additions decreases the ratio within minutes.

To follow the time course of the interaction, we used the ratio between the approximate maximum and minimum of the differential spectra (*R*  =  *I*_455 nm_/*I*_525 nm_), that correlates with the dipole potential variations. After the addition of each peptide, we observed a decrease on the dipole potential within the first minutes for erythrocytes (Fig. 3) and lymphocytes.

### Quantification of the membrane-peptide interaction for erythrocytes and PBMC

**Figure 4:**
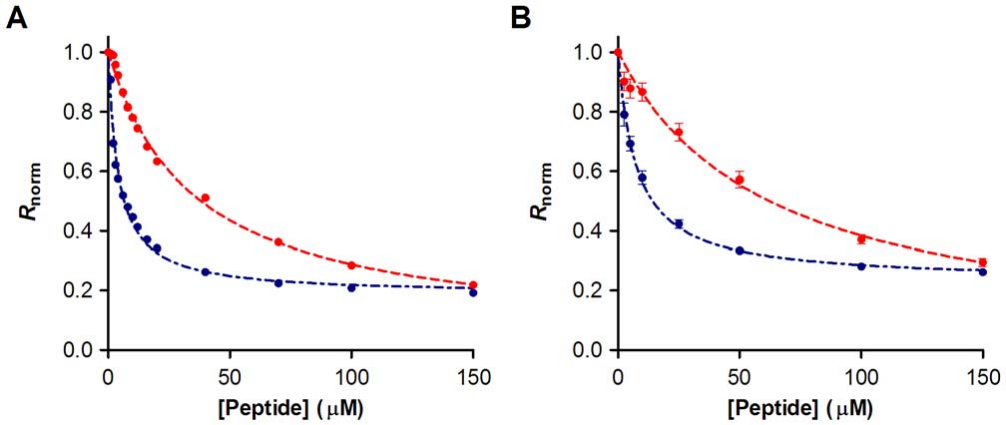
Peptide affinity towards erythrocytes and PBMC cell membranes. The dependence of the ratio on the enfuvirtide (red) and T-1249 (blue) concentrations for erythrocytes (A) and PBMC (B) was analyzed by a single binding site model (dashed lines) in order to quantify the dissociation constants (see Table 1). Ratio values were normalized for the initial value of zero peptide concentration. Plotted values represent the mean ± SEM (error bars not visible in erythrocytes due to small errors). N = 6 for erythrocytes and N = 7 for PBMC.

In order to quantify the affinity of the peptides to the cell membranes, we set to investigate the variation of the dipole potential as a function of peptide concentration (Fig. 4). The ratio *R* was measured for a range of concentrations of enfuvirtide and T-1249. The decrease of the potential as a function of concentration followed a hyperbolic curve. The data was analyzed according to a single binding site model [17] to yield dissociation constants (*K*_d_) for each peptide (Table 1). For both cell types, the *K*_d_ for T-1249 was much smaller (one order of magnitude), accounting for a higher preference for the cell membrane. The relative behavior of the peptide towards the erythrocyte and PBMC membrane seems to be maintained: the ratios *K*_d,ENF_/*K*_d,T-1249_ are similar for both cell type (8.5 and 8.1 for erythrocytes and PBMC, respectively).

**Table 1:** Quantification of peptide binding.

Cells	Peptide	*R*_min, norm_	*K*_d_ (µM)	*K*_d, ENF_/*K*_d, T-1249_
Erythrocytes	T-1249	−0.81±0.0063	4.16±0.13	8.5
	Enfuvirtide	−0.97±0.015	35.4±1.33	
PBMC	T-1249	−0.77±0.014	7.71±0.61	8.1
	Enfuvirtide	−1.0±0.071	62.2±10.2	

Comparison of the values of the dissociation constant, *K*_d_ and the parameter *R*_min_ obtained by non-linear regression with equation (1). The values represent mean ± SEM.

### Comparison of peptide-induced dipole changes in bio-mimetic lipid vesicles

**Figure 5:**
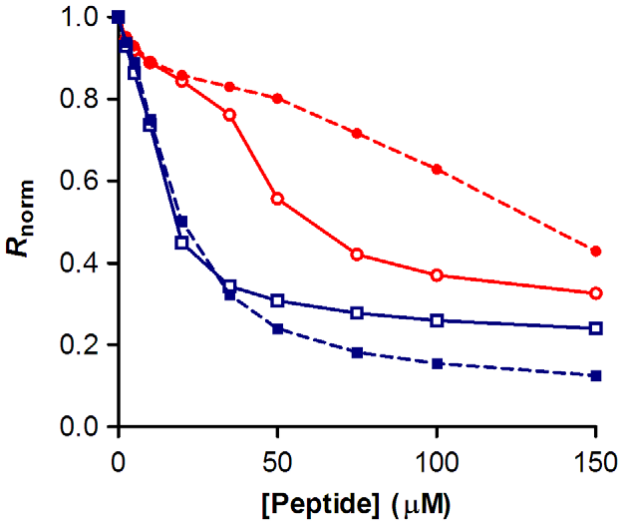
Interaction of the peptides with membrane model systems. The binding profiles for enfuvirtide (red) and T-1249 (blue) were obtained for large unilamellar vesicles composed of POPC (open symbols) and POPC/cholesterol 33% mol (filled symbols).

As stated earlier, interaction of enfuvirtide and T-1249 with lipid vesicles has been previously studied by means of their tryptophan residues fluorescence. For the sake of comparison with the data obtained for the interaction with blood cells membranes and in order to validate the comparison with previous data, we used di-8-ANEPPS labeled lipid vesicles to assess the interaction of these peptides, reported by changes on membrane dipole potential (Fig. 5). The lipid vesicles were composed entirely of POPC, to mimic most of the outer leaflets of mammalian cell membranes, in the liquid disordered state, or a mixture of POPC with cholesterol 33% mol, as an approximation to the membrane microdomains in the liquid ordered state (lipid rafts). This is especially relevant for the erythrocyte membranes, which are particularly cholesterol-rich [18]. Concerning the POPC vesicles, the binding of T-1249 was again more pronounced than for enfuvirtide (Fig. 5). When the vesicles are enriched with cholesterol, significant changes occur in terms of peptide association to the membrane. Enfuvirtide binds less extensively to membranes rich in cholesterol, while T-1249 increases the extension of membrane association (Fig. 5). This is in agreement with previous results obtained for lipid vesicles [12], in which the adsorption of T-1249 on cholesterol enriched domains was identified, in contrast to enfuvirtide.

## Discussion

Designing peptide drugs based in gp41 sequence to prevent viral entry into host cells has been in focus in the antiretroviral research field. Several factors related to their mode of action must be taken into account in order to optimize efficacy. For the first time, we addressed the interactions of HIV-1 fusion inhibitors with human cell membranes to better understand their membranotropic behavior *in vivo*. We made use of a dipole potential membrane probe, di-8-ANEPPS, to indirectly assess the membrane binding of enfuvirtide and T-1249 to erythrocytes and mononuclear leukocytes. These peptides decreased the membrane dipole potential of the two cell types, indicating that an interaction is occurring. Quantitatively, T-1249 was found to have approximately 8 times more affinity towards these cell membranes than enfuvirtide. Furthermore, studies with di-8-ANEPPS labeled POPC vesicles corroborated this by showing higher binding of T-1249 in comparison with enfuvirtide. Concerning POPC/cholesterol vesicles, the relative binding affinity between the two peptides was maintained. The presence of cholesterol reduced the binding extension of enfuvirtide and had an opposite effect in the case of T-1249, in agreement with our previous results that followed the interaction by tryptophan fluorescence [12]. Due to the fact that erythrocyte membranes contain significantly more cholesterol than PBMC [18] it could be expected that peptide binding characteristics for these two cell types would be more distinct. However, these composition differences seem not to be sufficient to change significantly the interaction behavior of the peptides, as we can see confronting POPC with POPC/cholesterol (33% mol) vesicles. Other factors such as the diversity of lipidic, proteic and glycidic components seem to superimpose to the effect of differences in cholesterol content. In fact, we checked if cholesterol depletion of erythrocytes membranes by methyl-β-cyclodextrin would reflect on changes in peptide binding and no significant alterations were detected (data not shown).

**Figure 6:**
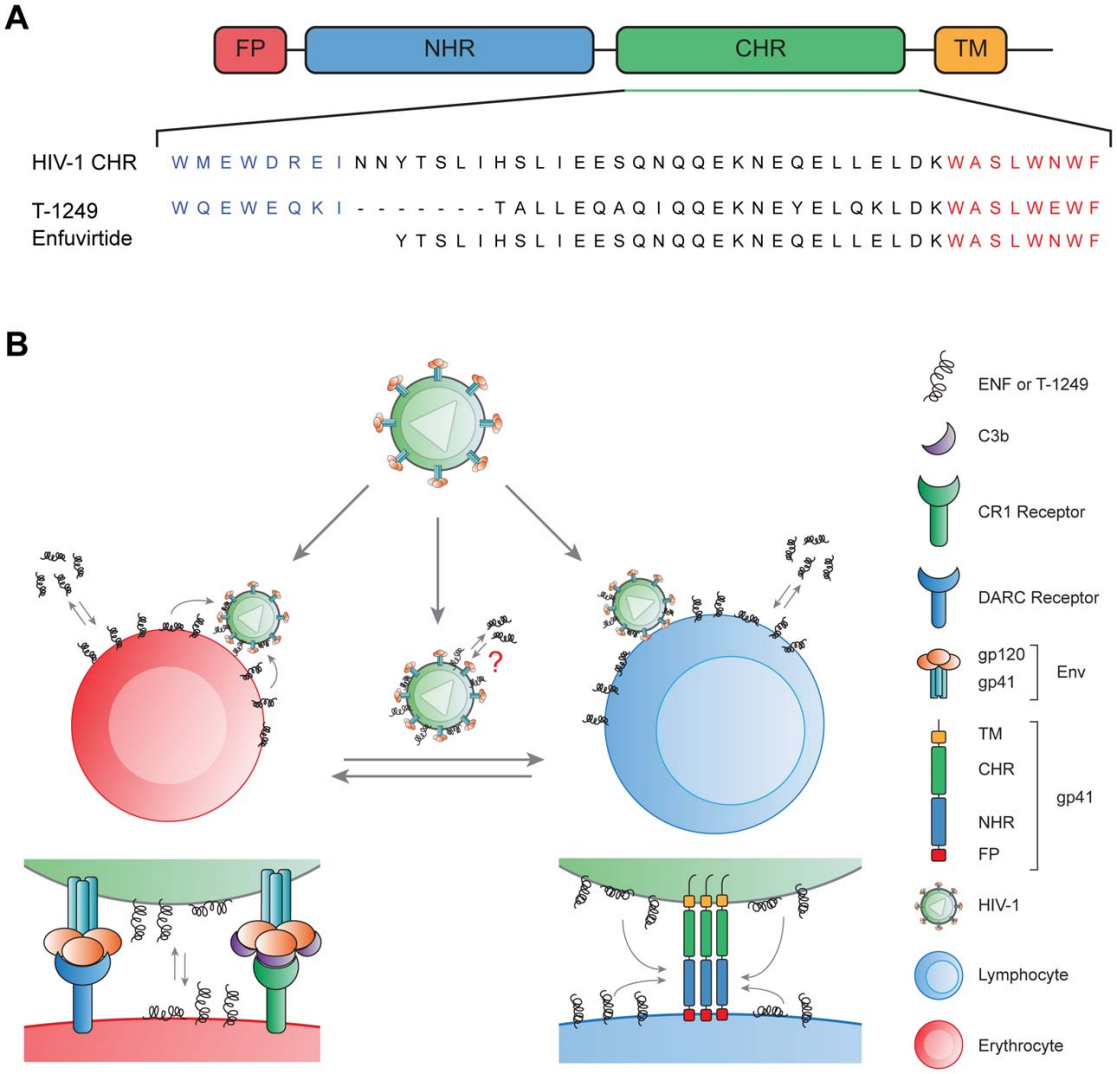
Proposed mode of action of enfuvirtide and T-1249 in circulation with blood cells. A) Sequence alignment of enfuvirtide and T-1249 with native gp41 (PDB code: 1ENV), showing the pocket binding domain (PBD) in blue and the putative lipid binding domain (LBD) close to the membrane proximal region in red. B) Peptides partition to the membranes of circulating blood cells creating a peptide-rich environment, when the virus interact both with erythrocytes and lymphocytes. HIV associates with erythrocytes via DARC receptor or complement opsonized virus via CR1. Exchanges of peptide between cells and virus and partition of peptide directly to the viral membrane can occur. When infecting the preferential CD4^+^ T cells, the membrane binding of peptide inhibitors should facilitate its interaction with the exposed conformation of gp41, preventing the 6-helix bundle formation. If the virus is internalized and fuses with the cell membrane at the endosome level, as recently described [27], the partition of the peptides to the membranes ensures that a considerable amount of peptides enters upon endocytosis in order to be available during the intracellular fusion event.

The membranotropism of these peptides can be explained in molecular terms by their structure and aminoacid sequence. It has been identified in the CHR and adjacent regions specific motifs that account for different roles in the interaction with NHR and membrane environment. Some of these regions also translate to the CHR derived peptides. Towards the N-terminus of CHR, there is a pocket binding domain (PBD, HIV-1_LAI_ 628–635) that binds to a hydrophobic groove in the NHR region [7]. It was established that this PBD was a crucial region for the binding of CHR and NHR in order to form the 6-helix bundle hairpin-like structure [7]. Another region identified in the far C-terminus of CHR is the lipid binding domain (LBD), a hydrophobic Trp-rich region by which CHR derived peptides can apparently bind to lipid membranes [19]. The sequence of enfuvirtide contains only the LBD while T-1249 was designed to contain both LBD and PBD (Fig. 6A). Thus, binding to cell and mimetic membranes is related to this putative LBD. Interestingly, by mutating the LBD region of enfuvirtide WNWF to ANAA, the antiviral activity of the peptide was abrogated [20] as well as its ability to bind to POPC vesicles [19]. Moreover, when an ANAA enfuvirtide mutant was octylated, its inhibitory activity was restored to a level similar to standard enfuvirtide [21]. This indicates the importance of the LBD for the efficacy of enfuvirtide, as the octylation of enfuvirtide also increased its potency. A similar result was obtained when enfuvirtide and ANAA mutant were expressed attached to a membrane protein in a T-helper cell line [22]. Another example comes from the addition of a cholesteryl group to C34, another peptide fusion inhibitor that lacks a LBP but contains the PBD sequence [23]. The potency of the derivatized C34 increased significantly; however, this effect was not observed when cholesterol was attached to enfuvirtide, which already has a LBD. TRI-999, another LBD lacking C-peptide, was synthesized with an attached C18 chain [24], showing improved efficiency over enfuvirtide.

All these findings that relate membrane binding to improved efficiency of the peptides mean that this is a major factor to take into account when rationally designing drugs of this type. Enfuvirtide and T-1249 have membranotropic behavior towards POPC lipid vesicles and now this study confirms that they both also interact with circulating blood cells. These peptides have a restricted temporal and spatial window of opportunity to bind to their molecular target: the NHR region of gp41 in its extended conformation when the virus and host cell are closely engaged. This makes aqueous diffusion of the peptide to the target less efficient. The capacity that these peptides have to bind to the cell membranes facilitates the delivery of the peptides to this confined environment, as some peptide is already locally present. Ultimately, the membrane can act as a “catalyst” to the binding reaction between the gp41 and the peptides, as it has been postulated in other receptor-ligand scenarios in the membrane environment [25]. Thus, for this case, the higher potency of T-1249 in relation to enfuvirtide seems to correlate with its higher membranes binding to the blood cells, particularly the lymphocytic fraction, where the preferential target cells are included. Then, its membrane binding capacity is possibly one of the factors that explain their relative potency (Fig. 6B). In terms of its structure and sequence, both peptides have the LBD sequence that accounts almost totally for enfuvirtide membrane binding. Nevertheless, T-1249 should have some other structural characteristic that account for its higher binding affinity. The PBD region additionally present on T-1249 is also a hydrophobic region and has 2 tryptophan residues (LBD has 3 Trp). This could account for a weak membrane binding domain. However, the membrane binding capacity of a C-peptide with an altered heptad repeat sequence and containing a PBD but not a LBD (PBD-4HR, similar do C34) is very poor, similar to a C-peptide that does not have any of these domains [26]. We could speculate that a possible, but not certain, synergistic action between the PBD and LBD domains present on T-1249 to increase its membranotropism. Another contributing factor could be the increased helicity of T-1249 in solution compared to enfuvirtide [12], a structural characteristic that favors insertion and stability in the membrane. Summarizing, T-1249 would be more effective due to: a) a LBD sequence motif, shared with enfuvirtide, that allows the peptide to anchor to the membrane as a platform to reach the target sequence in gp41 and explains part of its membranotropism; b) a PBD sequence motif that accounts for an enhanced gp41 binding and c) the higher overall affinity towards cell membranes of T-1249 (especially cholesterol-rich domains) not explained by the sum of the membrane binding effects of PBD and LBD.

Recently, a novel discovery about the HIV-1 entry into host cells challenged the current knowledge of viral-cell membrane fusion at the cell surface. Miyauchi *et al.* [27] showed that HIV-1 tends to prefer an endocytic route of entry and that fusion preferentially occurs at the endosomes. This would elicit protection to HIV from fusion inhibitors, as the surface exposition of the gp41 extended conformation is reduced. Hence, the capability of peptide fusion inhibitors to partition to the membrane and remain there while the virus is internalized, together with a cell membrane patch, can prove to be a decisive factor concerning their inclusion on the endosome, leading to increased antiretroviral efficacy and potency.

The relevance of peptide binding to erythrocytes is not to be diminished. In fact, HIV can be found associated with erythrocytes *in vivo* [28] and binds to erythrocyte membrane *in vitro* [29]. The virus can bind to erythrocytes *via* the DARC (Duffy Antigen Receptor for Chemokines) [30], [31] or *via* the complement receptor 1 (CR-1), that binds C3b or C3bi opsonized virus [32], [33]. Moreover, erythrocytes can mediate trans-infection of these bound viruses to other immune system cells that circulate in the blood and reside in the spleen [30], [32]. Interestingly, depending on which complement proteins are opsonizing the virus, they can direct the virus through various immune system cells that express different complement receptors, creating a dynamic circulation of the virus and facilitating its spreading through the body [34]. Therefore, we can speculate that by contacting with erythrocytes with pre-bound fusion inhibitor peptides, the virus itself can take peptide in its own membrane (Fig. 6B). In fact, Aloia *et al.* [35] found the lipid composition of the erythrocyte membrane to be similar to the HIV membrane itself; thus, peptide-viral membrane interaction seems to be very feasible. This peptide-membrane interaction can occur also for other circulating cells.

In a therapeutic context, several factors can influence the availability of the antiretroviral peptides in circulation. The drug is distributed to different components of the blood, namely, dissolved in the plasma, bound to plasma proteins (namely, albumin) and, as we established with this study, bound to cell membranes. The membrane-bound fraction would enhance the availability towards the molecular target in the virus, as explained earlier in this section, opposing to the fraction bound to plasma proteins, a factor that enhances peptide clearance. In conclusion, the dynamics between the peptide interaction among the different cell populations and with the virus, along with emerging new perspectives of HIV entry are complex factors that should the taken in consideration when rationally designing the next generation of fusion inhibitor peptides.

## Materials and Methods

### Isolation and labeling of human erythrocytes and PBMC

Human blood samples were obtained from healthy volunteer donors at the public blood bank Instituto Português do Sangue (Lisbon, Portugal), with their informed written consent. These samples were obtained under an institutional agreement between IPS Lisboa and Instituto de Bioquímica from Faculdade de Medicina da Universidade de Lisboa. This study was approved by the Ethics Committee of Faculdade de Medicina da Universidade de Lisboa. Donors were asked to give up to 10 mL of blood for research purposes additionally to their common blood donation of 450 mL to the blood bank. The blood collection was made anonymously as the data treatment resulting from the experiences made with these samples. The samples were drawn to K_3_EDTA anticoagulant tubes (Vacuette, Greiner Bio-one, Kremsmünster, Austria). For erythrocytes, the blood samples were centrifuged at 1200 *g* during 10 min to remove plasma and buffy-coat. The erythrocytes were washed at least two times with working buffer (HEPES 10 mM pH 7.4, in NaCl 150 mM, both from Sigma, St. Louis, MO, USA). For labeling, they were prepared at 1% hematocrit in working buffer supplemented with 0.05% (m/V) Pluronic F-127 (Sigma) and di-8-ANEPPS 10 µM (Molecular Probes, Invitrogen, Carlsbad, CA, USA). PBMC were isolated by density gradient using Ficoll-Paque Plus (GE Healthcare, Little Chalfont, UK), accordingly to manufacturer's instructions. Cells were counted in Cell-Dyn 1600 analyzer (Abbott, Abbott Park, IL, USA) and were prepared at 3000 cells/µL in Pluronic supplemented buffer with di-8-ANEPPS 3.3 µM. The suspensions of erythrocytes or PBMC with probe were incubated at room temperature with gentle agitation for 1 h. Unbound di-8-ANEPPS was removed by two wash cycles, with centrifugations at 1500 *g* for 5 min.

### Preparation and labeling of lipid vesicles

Large unilamellar vesicles (LUV) with ∼100 nm diameter, composed of pure POPC (Avanti Polar Lipids, Alabaster, AL, USA) or POPC with 33% mol cholesterol (Sigma), were obtained by extrusion of multi-lamellar vesicles (MLV) as described elsewhere [36]. Briefly, a chloroform dissolved lipid was dried in round-bottom flasks to obtain a film. MLV were obtained by dissolving the film in working buffer and subjecting to 8 freeze-thaw cycles. The suspension was extruded, first through a polycarbonate filter with 400 nm pores, followed by passages trough 100 nm pores filters in a custom made extruder. For cholesterol enriched vesicles, the extruder was heated by a water bath. Lipid vesicles suspensions with 500 µM of total lipid were incubated overnight with di-8-ANEPPS 10 µM, to ensure maximum incorporation of the probe.

### Fusion inhibitor peptides

The HIV-fusion inhibitors enfuvirtide (T-20) and T-1249 (kind gifts of Roche, Palo Alto, CA, USA) were prepared in working buffer at maximum stock concentration of 250 µM. These peptides were incubated at different concentrations with labeled erythrocytes (0.02% hematocrit), PBMC (100 cells/µL) or lipid vesicles (200 µM of total lipid) during 1 h for cells or 90 min for vesicles, with gentle agitation before measurements.

### Microscopy imaging

Erythrocyte microscopy images were obtained in a laser scanning confocal microscope Zeiss LSM 5 Live (Jena, Germany) using a water immersion objective Zeiss C-Apochromat 20× (1.20 numerical aperture). PBMC images were obtained in a Zeiss LSM510 Meta using an oil immersion objective Zeiss Plan-Apochromat 63× (1.4 numerical aperture). Di-8-ANEPPS was excited by a 488 nm laser (LP 505 filter for erythrocytes, BP 575–615 for PBMC). For the PBMC, Hoescht dye (Molecular Probes, Invitrogen) was also used for nucleus labeling, excited by a 405 nm laser (LP 420 filter).

### Membrane dipole potential assessed by di-8-ANEPPS

The membrane probe di-8-ANEPPS assesses dipole potential by shifting its excitation spectrum. Differential spectra for detecting these shifts are obtained by subtracting the excitation spectrum of labeled cells in the presence of peptide from the spectrum in its absence. Before subtraction the spectra were normalized to the integrated areas to reflect only spectral shifts. The differential spectra are waveform shaped, which amplitude directly correlates with the peak shifting magnitude, and hence, with the dipole potential variation. Blue-shifts will result in differential spectra in which a maximum comes at a lower wavelength than the maximum, and the opposite for red-shifts.

To qualitatively define a value for the spectral shift and hence, the dipole potential, a ratio was established from fluorescence intensities at two wavelengths on the sides of excitation spectrum peak [15], [37]. We chose them by taking the corresponding wavelength values for the minimum and the maximum of the differential spectra, defining the ratio *R* for this case as *I*_455 nm_/*I*_525 nm_ (*I*, fluorescence intensity). It was already established that an increase in the membrane dipole potential leads to a blue-shift in the membrane incorporated di-8-ANEPPS and, consequently, to an increase in the ratio [15], [37]. We also made a simple test for erythrocytes by adding 6-ketocholestanol (Sigma), an agent that increases dipole potential, and we observed the expected behavior of di-8-ANEPPS.

The variation of *R* with the peptide concentration was analyzed by a single binding site model [17], following equation 1, with the *R* values normalized for *R*_0_, the value at zero peptide concentration. *R*_min_ defines the asymptotic minimum value of *R* and *K*_d_ is the dissociation constant.
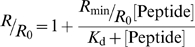
(Eq. 1)


### Fluorescence spectroscopy measurements

All the fluorescence spectroscopy measurements were made in a Varian Cary Eclipse (Mulgrave, Australia) fluorescence spectrophotometer. Membrane-bound di-8-ANEPPS excitation spectra and ratios were taken with emission at 670 nm in order to avoid membrane fluidity effects, as previously tested for lipid vesicles [38]. We also tested if this was observed in labeled erythrocytes (data not shown). The *R* values were measured through temperatures from 15°C to 50°C, with emissions at 580, 600 and 670 nm. The slope was lower for 670 nm, meaning a lower interference of fluidity effect at this wavelength. Excitation and emission slits for all measurements were 5 nm and 10 nm, respectively.
